# Analysis of compound heterozygotes reveals that the mouse floxed *Pax6*^*tm1Ued*^ allele produces abnormal eye phenotypes

**DOI:** 10.1007/s11248-016-9962-4

**Published:** 2016-05-30

**Authors:** Natalie J. Dorà, Aaron J. F. Crookshanks, Karen K. Y. Leung, T. Ian Simpson, John O. Mason, David J. Price, John D. West

**Affiliations:** 1 Genes and Development Group, Centre for Integrative Physiology, Biomedical Sciences, University of Edinburgh Medical School, Hugh Robson Building, George Square, Edinburgh, EH8 9XD UK; 2 Genes and Development Group, Centre for Integrative Physiology, Clinical Sciences, University of Edinburgh Medical School, Hugh Robson Building, George Square, Edinburgh, EH8 9XD UK; 3 Institute for Adaptive and Neural Computation, School of Informatics, University of Edinburgh, 10 Crichton Street, Edinburgh, EH8 9AB UK; 4 Biomathematics and Statistics Scotland, James Clerk Maxwell Building, Peter Guthrie Tait Road, Edinburgh, EH9 3FD UK

**Keywords:** Pax6, Floxed allele, Eye, Corneal epithelium, Keratin 12

## Abstract

Analysis of abnormal phenotypes produced by different types of mutations has been crucial for our understanding of gene function. Some floxed alleles that retain a neomycin-resistance selection cassette (neo cassette) are not equivalent to wild-type alleles and provide useful experimental resources. *Pax6* is an important developmental gene and the aim of this study was to determine whether the floxed *Pax6*^*tm1Ued*^ (*Pax6*^*fl*^) allele, which has a retained neo cassette, produced any abnormal eye phenotypes that would imply that it differs from the wild-type allele. Homozygous *Pax6*^*fl/fl*^ and heterozygous *Pax6*^*fl/*+^ mice had no overt qualitative eye abnormalities but morphometric analysis showed that *Pax6*^*fl/fl*^ corneas tended be thicker and smaller in diameter. To aid identification of weak effects, we produced compound heterozygotes with the *Pax6*^*Sey*-*Neu*^ (*Pax6*^−^) null allele. *Pax6*^*fl/*−^ compound heterozygotes had more severe eye abnormalities than *Pax6*^+*/*−^ heterozygotes, implying that *Pax6*^*fl*^ differs from the wild-type *Pax6*^+^ allele. Immunohistochemistry showed that the *Pax6*^*fl/*−^ corneal epithelium was positive for keratin 19 and negative for keratin 12, indicating that it was abnormally differentiated. This *Pax6*^*fl*^ allele provides a useful addition to the existing *Pax6* allelic series and this study demonstrates the utility of using compound heterozygotes with null alleles to unmask cryptic effects of floxed alleles.

**Electronic supplementary material:**

The online version of this article (doi:10.1007/s11248-016-9962-4) contains supplementary material, which is available to authorized users.

## Introduction

Analysis of genetic mutations has provided key insights in developmental genetics and an allelic series of different types of mutations provides a powerful way of studying gene function. Many new mouse alleles have been generated by conventional mutagenesis but some have also been produced as by-products when a neomycin-resistance selection cassette (neo cassette) is included in the construction of a floxed allele for Cre-*loxP* conditional knockout experiments. Retention of the neo cassette often reduces gene expression, sometimes causing hypomorphic phenotypes (Wassarman et al. 1997; Meyers et al. 1998; Nagy et al. 1998; Ashery-Padan et al. 2000; Levin and Meisler 2004; Walisser et al. 2004a, b; Moza et al. 2007; Kerr et al. 2008; Lin et al. 2008; Wang et al. 2011) but it may also increase expression of a reporter transgene (Scarff et al. 2003). Sometimes new alleles are deliberately generated in this way but an abnormal phenotype is usually an unwelcome side effect of constructing a floxed allele so the neo selection cassette is often excised.

*Pax6* is a semi-dominant gene with important roles in development of the eye, brain, nose, craniofacial tissues, pancreas, pituitary gland and pineal gland (Simpson and Price 2002; Favor et al. 2008). The floxed *Pax6*^*tm1Ued*^ allele (abbreviated to *Pax6*^*fl*^) was originally produced to analyse these roles using a conditional knockout approach (Simpson et al. 2009) but the possibility that it might produce an abnormal phenotype has not been investigated.

Homozygous *Pax6*^−*/*−^ and heterozygous *Pax6*^+*/*−^ phenotypes have been well characterised. The mouse small eye mutation, *Sey* (Roberts 1967) was identified as a *Pax6*^−^ null allele and renamed *Pax6*^*Sey*^ (Hill et al. 1991). Homozygous *Pax6*^−*/*−^ null mice, such as *Pax6*^*Sey/Sey*^ and *Pax6*^*Sey*-*Neu*/Sey-Neu^, die around the time of birth with no eyes, no nasal cavities and abnormal brain development (Hogan et al. 1986; Hill et al. 1991; Schmahl et al. 1993; Grindley et al. 1995).

In *Pax6*^+*/*−^ heterozygotes, the nasal cavities are unaffected, brain defects appear to be restricted to the subcommissural organ (Estivill-Torrus et al. 2001) but eyes show a wide range of abnormalities, including small size, iris hypoplasia, cataracts, a closed anterior chamber angle (irido-corneal angle) and corneal abnormalities that may include a persistent lens stalk, indicative of incomplete separation of the lens and corneal epithelium (Hogan et al. 1986; Hill et al. 1991; Grindley et al. 1995; van Raamsdonk and Tilghman 2000; Collinson et al. 2001; Baulmann et al. 2002). The adult mouse *Pax6*^+*/*−^ cornea shows progressive deterioration, leading to loss of transparency and this is probably caused partly by a stem cell deficiency. The corneal epithelium is thin and fragile with reduced keratin 12 expression, increased cell turnover, accumulation of goblet cells, abnormal cell movement and abnormal wound-healing (Ramaesh et al. 2003; Davis et al. 2003; Sivak et al. 2004; Collinson et al. 2004; Ramaesh et al. 2005, 2006; Leiper et al. 2006; Douvaras et al. 2013). The *Pax6*^+*/*−^ cornea is also invaded by sub-epithelial blood vessels with associated connective tissue (pannus) and inflammatory cells, which causes corneal opacities (Ramaesh et al. 2003).

The severity of *Pax6* mutant phenotypes may be affected both by stochastic effects and the genetic background (Pritchard 1973; Quinn et al. 1997; Ramaesh et al. 2009). For example, in heterozygous *Pax6*^+*/*−^ mice, eye size may vary between left and right eyes as well as among different mice (Roberts 1967; Clayton and Campbell 1968). Also, selection for a more permissive combination of modifier genes, present in gene pools of outbred mouse colonies, might explain why eye abnormalities in outbred mice may be more severe than for the same genotypes on more defined genetic backgrounds (Ramaesh et al. 2009). Examples of unusually severe adult *Pax6*^+*/*−^ eye phenotypes include highly abnormal lenses on an undefined mixed genetic background (Fig. 18 in Clayton 1970; Clayton and Campbell 1968) and adhesions and other mesenchymal abnormalities in outbred CD-1 mice (Kanakubo et al. 2006).

The *Pax6*^*fl*^ allele was produced by inserting a neo cassette, flanked by two *loxP* sites, into *Pax6* intron 4 (in sense orientation with respect to the endogenous *Pax6* locus) and another *loxP* site was inserted into intron 6. Thus, *Pax6* exons 5, 5a and 6, which encode the paired domain, were flanked by *loxP* sites. Exposure of this conditional null *Pax6*^*fl*^ allele to Cre-recombinase causes recombination of the *loxP* sites and deletion of exons 5, 5a and 6, which are needed for Pax6 function. Deletion of these exons is expected to cause a nonsense frame-shift mutation that converts the floxed *Pax6*^*fl*^ allele to the deleted *Pax6*^*Δ*^ form, which is functionally a null allele (Simpson et al. 2009).

Unlike *Pax6*^−*/*−^ and *Pax6*^+*/*−^, the *Pax6*^*fl/*+^, *Pax6*^*fl/fl*^ and *Pax6*^*fl/*−^ phenotypes have not been characterised. Despite the presence of the neo cassette in the *Pax6*^*fl*^ allele, homozygous *Pax6*^*fl/fl*^ mice were reported to be viable, fertile and phenotypically indistinguishable from wild-type mice (Simpson et al. 2009). Although the gross eye phenotype of *Pax6*^*fl/fl*^ homozygotes appeared to be normal, this was not investigated in detail and eye development is known to be particularly sensitive to both reduced and elevated Pax6 levels, resulting in a range of eye abnormalities (Hill et al. 1991; Schedl et al. 1996).

The aim of the present study was to use a more sensitive method to determine whether the *Pax6*^*fl*^ allele produced an abnormal eye phenotype. To compare the effects of a single *Pax6*^*fl*^ allele versus a single wild-type *Pax6*^+^ allele on eye phenotypes we crossed *Pax6*^*fl/*+^ heterozygotes with *Pax6*^+*/*−^ mice, heterozygous for the *Pax6*^*Sey*-*Neu*^ (*Pax6*^−^) null allele on the same genetically defined, inbred background. This revealed that eye phenotypes in *Pax6*^*fl/*−^ compound heterozygotes were more abnormal than those in *Pax6*^+*/*−^ heterozygous mice, implying that the *Pax6*^*fl*^ allele is not equivalent to the wild-type *Pax6*^+^ allele.

## Materials and methods

### Mice

Animal work was approved by the University of Edinburgh Ethical Review Committee and performed in accordance with UK Home Office regulations under project licenses PPL 60/3635 and PPL 60/4302. Heterozygous *Pax6*^+*/Sey*-*Neu*^ mice (abbreviated to *Pax6*^+*/*−^) were maintained by crossing to inbred CBA/Ca mice (N > 20 backcross generations) and genotyped by polymerase chain reaction (PCR) (Quinn et al. 1996). Heterozygous *Pax6*^*tm1Ued/*+^ (abbreviated to *Pax6*^*fl/*+^) mice (Simpson et al. 2009) on an outbred CD-1 genetic background were crossed to CBA/Ca (N = 8) and genotyped by PCR (Simpson et al. 2009). (Unlike CBA/J and some CD-1 mice, the CBA/Ca inbred strain does not carry the *Pde6b*^*rd1*^ retinal degeneration mutation, which can be confused with other retinal defects.) Adult and fetal mice were produced from *Pax6*^*fl/*+^ × *Pax6*^+*/*−^ and *Pax6*^*fl/*+^ × *Pax6*^*fl/*+^ crosses. The morning that a vaginal plug was found was defined as embryonic day (E) 0.5 and E14.5 fetuses were chilled in ice cold PBS, decapitated and tail tips used for genotyping. Adult mice were killed at 12 weeks by cervical dislocation following inhalation of gaseous isoflurane anaesthetic.

### Histology and morphometric measurements

Fetal heads were fixed in 4 % paraformaldehyde (PFA) overnight at 4 °C and processed to paraffin wax. For adults, left and right eyes were removed immediately, rinsed, dried and weighed to determine the mean eye mass. The mean eye and corneal diameters were both calculated from two orthogonal measurements of each eye using a Wild M5A dissecting microscope fitted with a calibrated measuring graticule in one eyepiece. Eyes were then fixed in 4 % PFA overnight at 4 °C and processed to paraffin wax. One adult eye was sectioned per mouse in an anterior–posterior plane to produce 7 µm sections through the cornea, lens and retina. To avoid adult lenses shattering the wax block was kept wet during sectioning. Fetal heads were sectioned anterior–posterior in a horizontal plane to produce 7 µm sections through the developing eye that included cornea, lens, and retina. Morphological features were compared in sections using standard haematoxylin and eosin (H & E) staining. Tissue sections of adult eyes were photographed and measured using a calibrated Zeiss Axiovision 4.8 digital camera system on a Zeiss Axioplan 2 compound microscope. The average thickness of the whole cornea, corneal stroma (plus endothelium) and corneal epithelium were determined from measurements at two places, near the centre of the cornea, in sections close to the mid-section in one eye per mouse and the number of corneal epithelial cell layers was counted.

### Immunohistochemical staining

Antibodies and methods used for immunohistochemical staining of Pax6, keratin 5, keratin 12 and keratin 19 were the same as those reported previously for mouse eyes (Dorà et al. 2014). Sections were dewaxed in Histoclear (Fisher Scientific), rehydrated through graded alcohols to water, incubated in 3 % hydrogen peroxide in methanol for 15 min. then washed in 0.1 % phosphate buffered saline-Tween (PBS-T). To unmask antibodies, slides were incubated in 0.01 M citrate buffer (pH 6.0), in a 95 °C water bath for 35 min. then allowed to cool for 20 min. and washed in PBS. Sections were treated with 10 % blocking serum (species according to secondary antibody), 0.1 % bovine serum albumin (BSA) in PBS for 1 h. at room temperature then incubated with the primary antibody, overnight at 4 °C. Slides were washed in PBS, incubated in blocking serum (10 min.) then secondary antibody (45 min. at room temperature), washed in PBS and incubated with avidin-biotin reagent (ABC RTU Vectastain, Vector Labs PK-7100). Antibody was then visualised with 3,3′-diaminobenzidine (DAB) stain (5.9 ml 20 mM Tris pH 7.6, 100 μl 50 mg/ml DAB, 1 μl H_2_O_2_). Finally, sections were lightly counterstained with haematoxylin, dehydrated and coverslips were mounted with DPX mounting medium (BDH from VWR). Control slides were treated with blocking serum in place of primary antibody but otherwise treated identically.

For Pax6, the primary mouse anti-Pax6 antibody (Developmental Studies Hybridoma Bank, University of Iowa; DSHB Cat# pax6, RRID:AB_528427) was diluted 1:500 and the secondary antibody (Vector labs BA-9200 biotinylated goat anti-mouse) was diluted 1:200. For keratin 5 (K5), the primary rabbit anti-K5 antibody (Abcam ab53121) was diluted 1:100 and the secondary antibody (Vector Labs BA-1000, biotinylated goat anti-rabbit IgG) was diluted 1:200. For keratin 12 (K12), the primary goat anti-K12 antibody (Santa Cruz Biotechnology sc-17101) was diluted 1:500 and the secondary antibody (Vector Labs BA-5000) biotinylated rabbit anti-goat IgG was diluted 1:200. For keratin 19 (K19), the primary rabbit anti-K19 antibody (Lifespan Biosciences LS-C3372) was diluted 1:200 and the goat anti-rabbit secondary antibody was the same as that used for K5.

### Statistical analysis

Minimum group sizes were guided by previous experience and power calculations, using standard deviations from comparisons of other genotypes (Ramaesh et al. 2009) to ensure sufficient power to detect, as significant (*P* < 0.05), mean eye mass differences of 5 mg. One-way analysis of variance (ANOVA), followed by Tukey’s multiple comparison tests, and non-parametric Kruskal–Wallis tests, followed by Dunn’s multiple comparison tests, were calculated using GraphPad Prism 5.0c (GraphPad Software Inc., San Diego, USA).

## Results

### Abnormal *Pax6*^*fl/*−^ eye phenotype

**Fig. 1:**
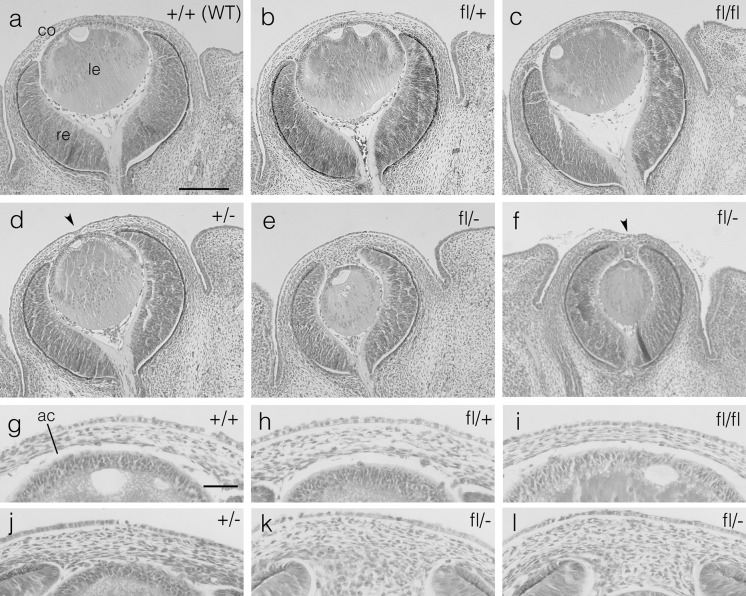
Histology of E14.5 fetal mouse eyes of five genotypes. **a**–**f** H & E stained sections showing whole eye morphology of **a** wild-type (WT) *Pax6* ^+*/*+^ (+/+), **b** *Pax6* ^*fl/*+^(fl/+), **c** *Pax6* ^*fl/fl*^ (fl/fl), **d** *Pax6* ^+*/*−^ (+/−) and **e**, **f** *Pax6* ^*fl/*−^ (fl/−) fetal eyes. The *arrowheads* in **d** and **f** indicate indentations in the corneas caused by persistent lens stalks (lens-corneal plugs). **g**–**l** Histology of anterior of eye of **g** +/+, **h** fl/+, **i** fl/fl, **j** +/− and **k**, **l** fl/− fetal eyes. Figures show representative eyes from 4 fetuses that were sectioned for each genotype. Different fl/− eyes are shown in **e**, **f**, **k** and **l**. *Scale bars* **a** (for **a**–**f**) = 200 μm; **g** (for **g**–**l**) = 50 µm. *ac* anterior chamber, *co* cornea, *le* lens, *re* retina

**Fig. 2:**
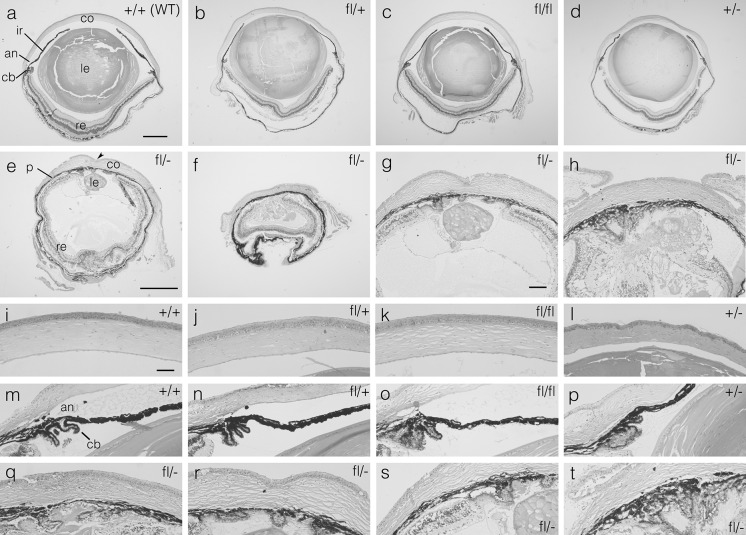
Histology of 12-week adult mouse eyes of five genotypes. **a**–**f** H & E stained sections showing whole eye morphology of **a** wild-type (WT) *Pax6* ^+*/*+^ (+/+), **b** *Pax6* ^*fl/*+^(fl/+), **c** *Pax6* ^*fl/fl*^ (fl/fl), **d** *Pax6* ^+*/*−^ (+/−) and **e**, **f** *Pax6* ^*fl/*−^ (fl/−) eyes. Eyes in **e** and **f** are small and are shown at a greater magnification than eyes in **a**–**d**. The *arrowhead* in **e** indicates an indentation in the cornea, caused by a persistent lens stalk. **g**, **h** Histology of anterior of fl/− eyes showing lens abnormalities. **i**–**l** Histology of central cornea of **i** +/+, **j** fl/+, **k** fl/fl and **l** +/− eyes. **m**–**p** Histology of ciliary body and irido-corneal angle (anterior chamber angle) of **m** +/+, **n** fl/+, **o** fl/fl and **p** +/−, showing closed angle in **p**. **q**–**t** Histology of anterior of fl/− eyes showing pigmented presumptive iris tissue adhering to the central cornea **q**, **r** and peripheral cornea **s**, **t**. Different views of the same fl/− eye are shown in **e**, **g**, **r** and **s** but they are not all from the same section. Different views of another fl/− eye are shown in **h** and **t**. Figures show representative eyes from 4 (*Pax6* ^*fl/fl*^) or 5 mice (other 4 genotypes) that were sectioned (1 eye per mouse). *Scale bars* **a** (for **a**–**d**) and **e** (for **e**, **f**) = 500 μm; **g** (for **g**, **h**) = 100 µm. **i** (for **i**–**t**) = 50 µm; *an* irido-corneal angle, *cb* ciliary body, *co* cornea, *ir* iris, *le* lens, *p* pigmented presumptive iris tissue, *re* retina

*Pax6*^*fl/*+^ and *Pax6*^+*/*−^ mice were crossed to produce offspring of four genotypes: *Pax6*^+*/*+^ (wild-type, WT), *Pax6*^*fl/*+^, *Pax6*^+*/*−^ and *Pax6*^*fl/*−^. Homozygous *Pax6*^*fl/fl*^ mice, on a similar, predominantly CBA/Ca genetic background, were produced in a separate *Pax6*^*fl/*+^ × *Pax6*^*fl/*+^ cross. Phenotypes of *Pax6*^+*/*+^*, Pax6*^*fl/*+^, *Pax6*^*fl/fl*^, *Pax6*^+*/*−^ and *Pax6*^*fl/*−^ eyes are shown in Figs. 1 and 2. For both E14.5 fetuses and 12-week-old adults, heterozygous *Pax6*^*fl/*+^ and homozygous *Pax6*^*fl/fl*^ eyes appeared to be grossly unaffected and qualitatively equivalent to *Pax6*^+*/*+^ (wild-type). However, *Pax6*^*fl/*−^ compound heterozygous eyes were clearly more severely affected than *Pax6*^+*/*−^ heterozygotes. At E14.5 (Fig. 1), the most obvious abnormalities of heterozygous *Pax6*^+*/*−^ eyes were that the anterior chamber was not yet formed, as reported previously (Ramaesh et al. 2003), and some had an indentation in the middle of the cornea, indicative of a persistent lens stalk (lens-corneal plug). In contrast, lenses of E14.5 *Pax6*^*fl/*−^ compound heterozygotes eyes were small and usually did not protrude beyond the anterior of the optic cup. The anterior chamber had not formed and mesenchymal cells extended from the cornea into the anterior of the optic cup and coated the anterior of the lens. Persistent lens stalks, involving the surface ectoderm, were only seen in some of the eight E14.5 *Pax6*^*fl/*−^ eyes that were examined (e.g. Fig. 1f).

At 12 weeks, *Pax6*^+*/*+^, *Pax6*^*fl/*+^ and *Pax6*^*fl/fl*^ eyes all appeared grossly normal (Fig. 2a–c, i–k, m–o) apart from some common processing artefacts, which included separation of the retinal pigment epithelium from the neural retina and some lens damage. As described elsewhere (Ramaesh et al. 2003), *Pax6*^+*/*−^ eyes (Fig. 2d, l, p) showed a range of abnormalities, including slightly reduced size, hypoplastic irises, thinner corneal epithelium and sometimes a lens-corneal plug (persistent lens stalk). Adult *Pax6*^+*/*−^ eyes had an anterior chamber but, in some cases, the irido-corneal angle (anterior chamber angle) was closed, as reported previously (Baulmann et al. 2002). However, *Pax6*^*fl/*−^ eyes were much smaller and more severely affected (Fig. 2e–h, q–t). They had no ciliary body, anterior chamber or irido-corneal angle and a layer of pigmented tissue, with no pupil opening, adhered to the corneal endothelium. This is presumed to be an abnormal iris but a persistent pupillary membrane could also be present, as described for *Pax6*^*Leca4/*+^ heterozygotes (Ramaesh et al. 2009). It is also possible that this is at least partly derived from the retinal pigment epithelium but we did not use markers to attempt to identify the origin of the pigmented layer. The *Pax6*^*fl/*−^ lens was small and dysplastic with vacuoles and sometimes adhesions to pigmented, presumptive iris tissue anteriorly and/or the retina posteriorly. In some *Pax6*^*fl/*−^ eyes a lens-corneal plug was present. The retina was severely dysplastic with, irregular layers, folds and rosettes (Fig. 2e) but the retinal pigment epithelium, adjacent to the neural retina, appeared grossly normal.

**Fig. 3:**
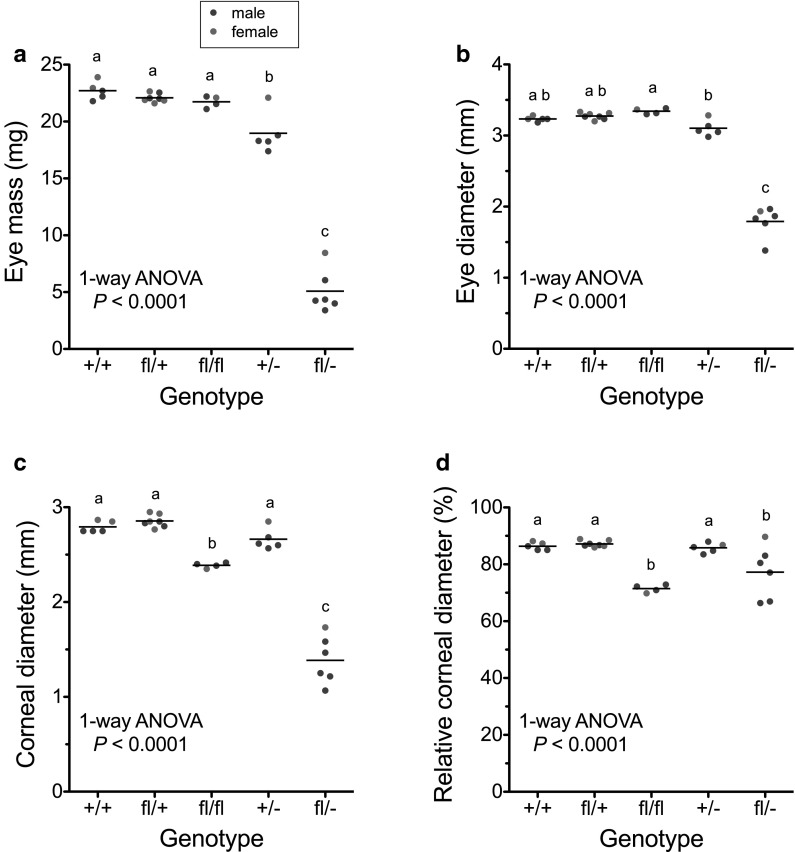
Comparisons of eye and corneal sizes for 12-week adult mice of five genotypes. Each point is the mean of *left* and *right* eyes of one mouse and the horizontal bars are group means for **a** eye mass, **b** eye diameter, **c** corneal diameter and **d** relative corneal diameter (corneal diameter as a percentage of eye diameter). The numbers of mice per group were 5 +*/*+, 7*fl*/+, 4*fl*/*fl*, 5 +*/*− and 6*fl*/−. Groups were compared by 1-way analysis of variance (ANOVA; results shown on figure) and Tukey’s multiple comparison tests. Genotypes, with only different letters above the scatter plots, differ significantly (*P* < 0.05). Genotypes, with any shared letters above the scatter plots, do not differ significantly. Sexes are coloured differently (*red* female, *blue* male) but were not analysed separately. (Color figure online)

Quantitative comparisons of eye mass confirmed that heterozygous *Pax6*^+*/*−^ eyes were significantly smaller than wild-type *Pax6*^+*/*+^ eyes (*P* < 0.001) but compound heterozygous *Pax6*^*fl/*−^ eyes were even smaller. *Pax6*^*fl/*−^ eye mass and diameter were significantly smaller than the other four genotypes (*P* < 0.001 in each case) but *Pax6*^*fl/*+^ and *Pax6*^*fl/fl*^ genotypes did not differ significantly from *Pax6*^+*/*+^ (Fig. 3a, b). The corneal diameter of *Pax6*^*fl/*−^ compound heterozygotes was smaller than the other four genotypes (*P* < 0.001 in each case) but it was also significantly smaller in *Pax6*^*fl/fl*^ than *Pax6*^+*/*+^ (*P* < 0.01), *Pax6*^*fl/*+^ (*P* < 0.001) and *Pax6*^+*/*−^ (*P* < 0.05), as shown in Fig. 3c. Furthermore, the relative corneal diameter (expressed as a percentage of eye diameter) was significantly smaller for *Pax6*^*fl/*−^ than *Pax6*^+*/*+^ (*P* < 0.05), *Pax6*^*fl/*+^ (*P* < 0.01) and *Pax6*^+*/*−^ (*P* < 0.05) but not *Pax6*^*fl/fl*^ (Fig. 3d). The *Pax6*^*fl/fl*^ relative corneal diameter was also significantly less than *Pax6*^+*/*+^, *Pax6*^*fl/*+^ and *Pax6*^+*/*−^ (*P* < 0.001 in each case).

Morphometric measurements were also used to compare corneal thickness but variation within groups was relatively high and, as group sizes were small, these results are considered preliminary and are presented in the graphs included as Supplementary Fig. S1. Despite the small group sizes (thus low statistical power), there was also a clear trend for *Pax6*^*fl/fl*^ eyes to have thicker corneas than *Pax6*^+*/*+^. Differences between these two genotypes were significant for the thickness of the corneal epithelium (*P* < 0.05) and whole cornea (*P* < 0.01) but not for the thickness of the corneal stroma (plus the thin corneal endothelium) or the number of corneal epithelial layers. *Pax6*^+*/*−^ mice are known to have thin and fragile corneal epithelia (Ramaesh et al. 2003) and the mean number of corneal epithelial cell layers was lowest for *Pax6*^+*/*−^ and *Pax6*^*fl/*−^. However, for the small samples analysed here, only *Pax6*^*fl/*−^ and *Pax6*^*fl/fl*^ differed significantly (*P* < 0.05).

**Fig. 4:**
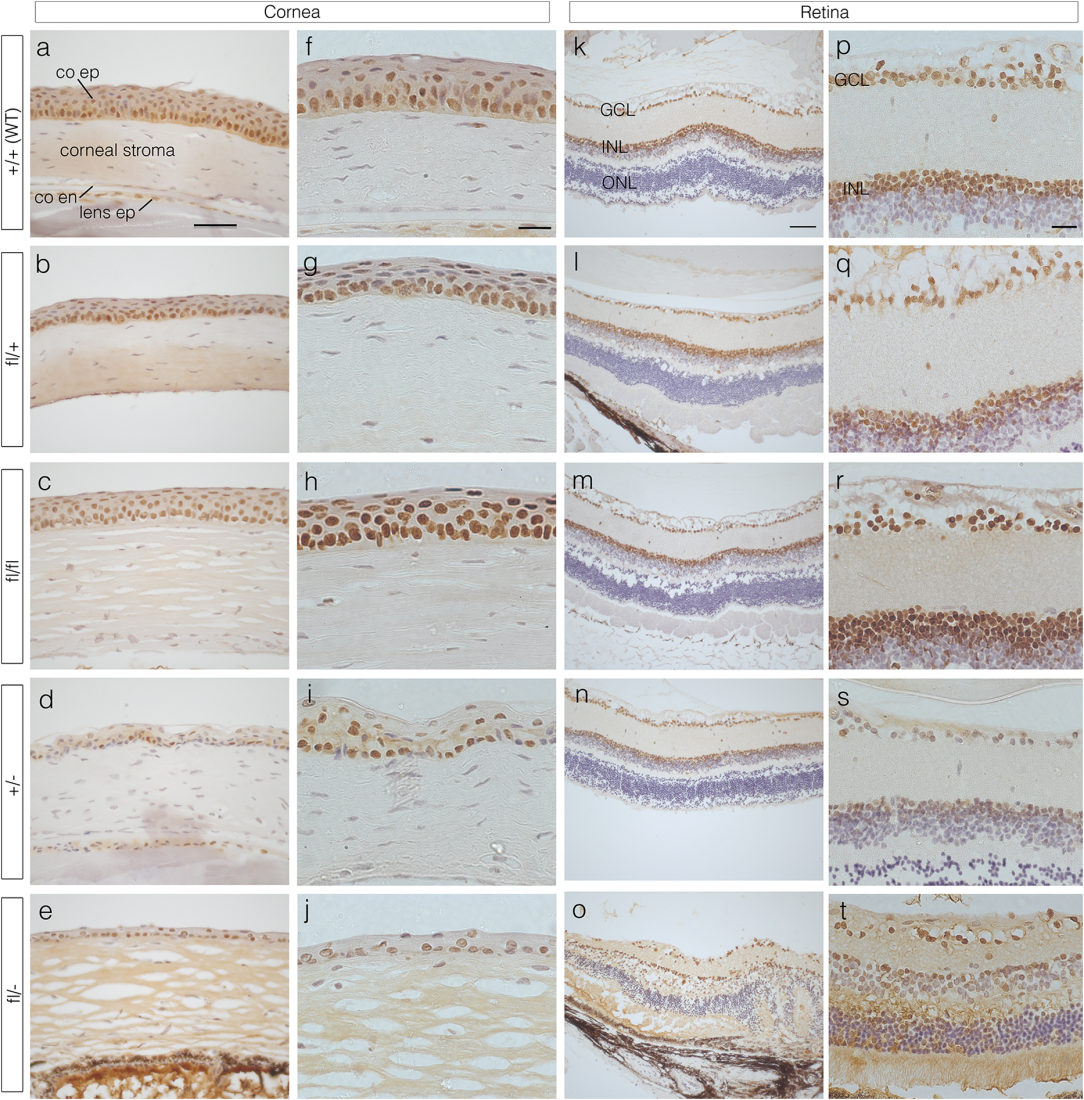
Pax6 immunohistochemistry in corneal epithelia and retinas from 12-week adult mouse eyes of five genotypes. Pax6 immunostaining is shown by the brown DAB endpoint and sections were counterstained with haematoxylin. **a**–**j** Corneal epithelium; **k**–**t** retina. The lens epithelium is also visible in **a** and **d**. **a**, **f**, **k**, **p** Wild-type (WT) *Pax6* ^+*/*+^ (+/+), **b**, **g**, **l**, **q** *Pax6* ^*fl/*+^(fl/+), **c**, **h**, **m**, **r** *Pax6* ^*fl/fl*^ (fl/fl), **d**, **i**, **n**, **s** *Pax6* ^+*/*−^ (+/−) and **e**, **j**, **o**, **t** *Pax6* ^*fl/*−^ (fl/−) mice. *Scale bars* for columns **a**–**e** and **k**–**o**, 50 μm; **f**–**j** and **p**–**t**, 20 µm. *co* cornea, *en* endothelium, *ep* epithelium, *GCL* ganglion cell layer, *INL* inner nuclear layer, *ONL* outer nuclear layer

Immunohistochemical staining for Pax6 was positive in the lens (not shown), corneal epithelium and retina of all 5 genotypes (Fig. 4). Although nuclear staining appeared to be stronger in *Pax6*^+*/*+^, *Pax6*^*fl/*+^ and *Pax6*^*fl/fl*^ than *Pax6*^+*/*−^ and *Pax6*^*fl/*−^ sections, Pax6 levels were not compared quantitatively.

### Abnormal expression of corneal epithelial markers in *Pax6*^*fl/*−^ mice

**Fig. 5:**
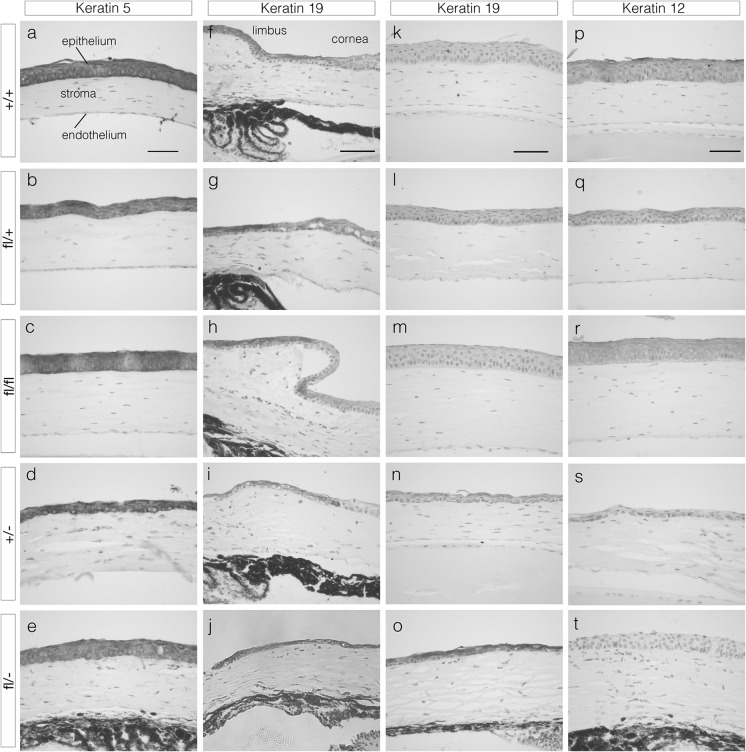
Keratin 5, 19 and 12 immunohistochemistry of corneal epithelia from 12-week adult mouse eyes of five genotypes. Immunostaining is shown by the brown DAB endpoint and sections were counterstained with haematoxylin. **a**–**e** Keratin 5 immunohistochemistry in the central cornea. The three cellular layers of the cornea are labelled in **a**. **f**–**o** Keratin 19 immunohistochemistry in the limbus and peripheral cornea **f**–**j** and central cornea **k**–**o**. **p**–**t** Keratin 12 immunohistochemistry in the central corneas. Genotypes: **a**, **f**, **k**, **p** *Pax6* ^+*/*+^ (+/+), **b**, **g**, **l**, **q** *Pax6* ^*fl/*+^(fl/+), **c**, **h**, **m**, **r** *Pax6* ^*fl/fl*^ (fl/fl), **d**, **i**, **n**, **s** *Pax6* ^+*/*−^ (+/−) and **e**, **j**, **o**, **t** *Pax6* ^*fl/*−^ (fl/−). *Scale bars* for each column, 50 μm

Keratin 5 (K5) is normally present in the mouse corneal epithelium, conjunctiva and the limbal epithelium, which is a junctional zone between these two tissues (Byrne and Fuchs 1993; Douvaras et al. 2012). Strong K5 immunostaining was present in these three ocular surface epithelia in all five genotypes and the central corneal epithelium is illustrated in Fig. 5a–e.

Keratin 19 (K19) is a marker of the mouse limbal epithelium and conjunctiva but not the central corneal epithelium (Yoshida et al. 2006). The limbal and peripheral corneal epithelia of all five genotypes stained positive for K19 (Fig. 5f–j). In the central corneal epithelia, K19 immunostaining was weak or absent in *Pax6*^+*/*+^*, Pax6*^*fl/*+^ and *Pax6*^*fl/fl*^ genotypes (Fig. 5k–m), patchy in *Pax6*^+*/*−^ (Fig. 5n) but stronger and clearly above background levels in *Pax6*^*fl/*−^ compound heterozygotes (Fig. 5o).

Keratin 12 (K12) is a specific corneal epithelial marker (Liu et al. 1993) and K12 staining was present in the central corneal epithelia of *Pax6*^+*/*+^*, Pax6*^*fl/*+^ and *Pax6*^*fl/fl*^ eyes (Fig. 5p–r). Some weak and patchy staining was present in the *Pax6*^+*/*−^ corneal epithelium (Fig. 5s) but K12 staining was absent from the *Pax6*^*fl/*−^ compound heterozygous corneal epithelium (Fig. 5t).

## Discussion

Compound heterozygous *Pax6*^*fl/*−^ mice had much more severe eye abnormalities than *Pax6*^+*/*−^ heterozygotes and expression of K12 and K19 were also more severely affected in *Pax6*^*fl/*−^ corneas. K12 is normally only expressed in the corneal epithelium and is regulated by Pax6 (Liu et al. 1993; Shiraishi et al. 1998). Conversely, K19 is normally expressed in the mouse limbal epithelium and conjunctiva but not the corneal epithelium (Yoshida et al. 2006). Consistent with previous reports, the *Pax6*^+*/*−^ corneal epithelium showed weak, patchy and sparse K12 staining (Ramaesh et al. 2003, 2005) and patchy K19 staining (Douvaras et al. 2013). Although K12 and K19 immunostaining was normal in the *Pax6*^*fl/*+^ and *Pax6*^*fl/fl*^ ocular surface, it was even more abnormal in *Pax6*^*fl/*−^ than in *Pax6*^+*/*−^. The more strongly positive K19 staining and absence of K12 in the *Pax6*^*fl/*−^ central corneal epithelium suggests that it is abnormally differentiated. However, we did not determine whether cells in the *Pax6*^*fl/*−^ corneal epithelium expressed other specific limbal or conjunctival markers or test for hallmarks of conjunctivilisation. Together, the morphology and corneal immunohistochemistry results for *Pax6*^+*/*−^ and *Pax6*^*fl/*−^ eyes imply that the floxed *Pax6*^*fl*^ allele is not equivalent to the wild-type *Pax6*^+^ allele.

The observations that corneas in *Pax6*^*fl/fl*^ homozygotes were thicker and had a smaller diameter than normal indicates that an abnormal *Pax6*^*fl*^ phenotype is detectable in *Pax6*^*fl/fl*^ homozygotes as well as *Pax6*^*fl/*−^ compound heterozygotes but the *Pax6*^*fl/fl*^ phenotype is much less severe. Although *Pax6*^*fl/fl*^ homozygotes and *Pax6*^+*/*+^ wild-type mice were produced by different genetic crosses, they were on very similar genetic backgrounds. Nevertheless, as sample sizes were small and corneal stromal thickness can be affected by histological processing, this evidence for an abnormal *Pax6*^*fl/fl*^ corneal phenotype should be considered preliminary. Apart from the subtle corneal differences revealed by morphometrics, there were no major qualitative morphological ocular abnormalities or aberrant K12 or K19 immunostaining in the corneal epithelium of *Pax6*^*fl/*+^ or *Pax6*^*fl/fl*^ mouse eyes. The phenotypic evidence suggests that *Pax6*^*fl*^ is an abnormal allele with a minor effect on corneas of *Pax6*^*fl/fl*^ homozygotes but no effect on *Pax6*^*fl/*+^ heterozygotes.

Most of the phenotypic abnormalities can be ordered as: −*/*− (anophthalmic; not shown) >*fl/*− > +*/*− > *fl/fl* > *fl/*+ = +*/*+ (normal). Although we have not investigated the molecular mechanisms, it is tempting to speculate how the different genotype-phenotype correlations could arise. For example, if this rank order of decreasing phenotype severity were caused by an equivalent rank order of increasing Pax6 levels, this would predict that the floxed *Pax6*^*fl*^ allele causes a relatively small quantitative reduction in Pax6 level, such that *Pax6*^*fl/fl*^ homozygotes have more Pax6 than *Pax6*^+*/*−^.

The above phenotype severity rank order is consistent with the differences in eye size but it does not fit so well with the results for corneal diameter or corneal epithelial thickness. However, the floxed *Pax6*^*fl*^ allele might affect different tissues in different ways. Although the *Pax6*^−^ null allele only affects Pax6 quantitatively, *Pax6*^*fl*^ might cause both a quantitative effect and a qualitative difference in Pax6 protein or a difference in the ratio of Pax6 isoforms. Any such effects would be expected to be most pronounced in *Pax6*^*fl/fl*^ and *Pax6*^*fl/*−^ genotypes, where there was no wild-type allele, and might affect *Pax6*^*fl/*+^ to a lesser extent but would not affect *Pax6*^+*/*−^ or *Pax6*^+*/*+^. This is consistent with the smaller relative corneal diameter seen in *Pax6*^*fl/fl*^ and *Pax6*^*fl/*−^ genotypes. In principle, the results for corneal epithelial thickness could be explained by an interaction between two opposing effects. Low Pax6 levels produce a thin corneal epithelium in *Pax6*^+*/*−^ heterozygotes (Davis et al. 2003; Ramaesh et al. 2003) and this may also be the predominant effect in *Pax6*^*fl/*−^ heterozygotes. However, if Pax6 levels are higher, an altered Pax6 protein (or altered isoform ratio) might have the opposite effect in *Pax6*^*fl/fl*^ homozygotes but not in *Pax6*^*fl/*+^ heterozygotes where wild-type protein is also present. Although such speculations, based on phenotypes, are useful for generating hypotheses, molecular investigations will be required to test these and other possibilities.

In contrast to the mildly abnormal *Pax6*^*fl/fl*^ and wild-type *Pax6*^*fl/*+^ phenotypes reported here, a more overtly abnormal phenotype was reported for mice carrying a different floxed *Pax6* allele (with the neo cassette in the intron between exons 6 and 7) and this was corrected by removing the neo cassette (cited in Ashery-Padan et al. 2000). Abnormal phenotypes have been reported for floxed alleles of other genes with retained neo cassettes (see Introduction) and some aberrant phenotypes have been attributed to altered gene expression caused by cryptic splice sites in the neo cassette (Nagy et al. 1998; Meyers et al. 1998). The retained neo cassette may also affect Pax6 expression in the floxed *Pax6*^*fl*^ allele, at least in the eye. K12 is regulated by Pax6 (Shiraishi et al. 1998; Liu et al. 1999) and, in humans, the Pax6(5a) isoform is known to be critical (Sasamoto et al. 2016). In principle, therefore, both the abnormal *Pax6*^*fl/*−^ eye morphology and the absence of K12 immunostaining in the *Pax6*^*fl/*−^ corneal epithelium could be mediated via direct effects on Pax6, such as reduced levels or altered ratios of different isoforms. However, we have not investigated the mechanisms that underlie the abnormal *Pax6*^*fl/*−^ phenotype.

Cryptic hypomorphic phenotypes have been unmasked for floxed alleles (*fl*) of *Nodal* and *Smad2* with retained neo or β-geo selection cassettes, using compound heterozygotes with null alleles (Lowe et al. 2001; Liu et al. 2004). Like *Pax6*^*fl*^, these were identified as abnormal alleles because *fl/null* compound heterozygotes had a more severe phenotype than +*/null* heterozygotes, even though *fl/fl* homozygotes appeared to be normal. The unmasking of cryptic hypomorphic phenotypes produced by floxed alleles of *Nodal*, *Smad2* and now *Pax6*, using compound heterozygotes with null alleles, demonstrates that this approach is likely to be useful for identifying other floxed alleles with weak effects that are otherwise difficult to detect.

The availability of different *Pax6* alleles provides a useful resource for the analysis of the roles of Pax6 in various tissues because *Pax6*^−^ null alleles alone are not always sufficiently informative. The anophthalmia and early lethality of *Pax6*^−*/*−^ homozygous mean that this genotype is only useful for early developmental stages unless used in mouse chimaeras (Quinn et al. 1996) and the *Pax6*^+*/*−^ genotype has only minor effects in tissues other than the eye. However, by using combinations of different mouse *Pax6* alleles, different phenotypes can be produced to suit the experimental requirements. Early studies showed that the phenotypes of heterozygous *Pax6*^+*/*−^ mice were similar to some human inherited conditions caused by *PAX6* mutants, including aniridia and Peters’ anomaly (Hill et al. 1991; Ton et al. 1991; Glaser et al. 1994; Hanson et al. 1994; Nishida et al. 1995; Davis et al. 2003; Ramaesh et al. 2003). The availability of a wider array of different mouse *Pax6* alleles may also provide models for some atypical human phenotypes that are caused by *PAX6* missense mutations (Hanson et al. 1999; Hingorani et al. 2012).

A large allelic series of mouse *Pax6* mutants has been assembled, mainly from conventional mutagenesis screens (Roberts 1967; Hogan et al. 1986; Hill et al. 1991; Lyon et al. 2000; Favor and Neuhauser-Klaus 2000; Favor et al. 2001; Thaung et al. 2002; Favor et al. 2008, 2009; Puk et al. 2013). As already noted, *Pax6*^−*/*−^ null homozygotes are anophthalmic and die around birth and heterozygotes have significant ocular defects including microphthalmia (Hill et al. 1991; Grindley et al. 1995). Four hypomorphic alleles (*Pax6*^*coo*p^, *Pax6*^*4Neu*^, *Pax6*^*7Neu*^ and *Pax6*^*132*-*14Neu*^) with less severe phenotypes have already been produced in mutagenesis experiments (Lyon et al. 2000; Favor et al. 2001, 2008). However, none of these is equivalent to the *Pax6*^*fl*^ allele reported here. *Pax6*^*coo*p^, *Pax6*^*4Neu*^ and *Pax6*^*7Neu*^ have more severe phenotypes. Homozygotes are anophthalmic and die perinatally while heterozygotes have significant overt eye abnormalities. *Pax6*^*132*-*14Neu*^ has a weaker effect than *Pax6*^*coo*p^, *Pax6*^*4Neu*^ or *Pax6*^*7Neu*^ but a stronger effect than *Pax6*^*fl*^. Homozygous *Pax6*^*132*-*14Neu/132*-*14Neu*^ mice are viable and fertile but eye defects are more severe than in *Pax6*^+*/*−^ heterozygotes and, although some *Pax6*^*132*-*14Neu/*+^ heterozygotes have no overt eye defects, others have a range of relatively minor abnormalities.

Two other alleles affect specific Pax6 isoforms. Mice heterozygous for a targeted deletion of the Pax6(5a) isoform develop iris hypoplasia and homozygotes have additional minor defects of the iris, retina, lens and corneal stroma but eye size is not reduced (Singh et al. 2002). Unlike the *Pax6*^*fl/*−^ phenotype, however, no ocular defects developed until after birth. The T(2;14)1GSo reciprocal translocation disrupts Pax6 regulation, causing elevated expression of the Pax6 P1’ isoform that results in thickening of the corneal epithelium and stroma (Elso et al. 2013). Further work is required to determine whether the floxed *Pax6*^*fl*^ allele shares features with T(2;14)1GSo translocation mice, which might predict the involvement of altered ratios of the Pax6 P1’ isoform.

Existing mouse hypomorphic alleles have already proved useful for analysis of Pax6 gene function in eye development. Combinations of *Pax6*^*4Neu*^, *Pax6*^*7Neu*^, *Pax6*^*132*-*14Neu*^ and other *Pax6* alleles in various compound heterozygotes have produced a range of ocular phenotypes that have been ranked and grouped into three phenotype classes (Favor et al. 2008). *Pax6*^*fl*^ now provides a novel addition to the existing *Pax6* allelic series that, in combination with other alleles, should be useful for refining these phenotype classifications.

## Electronic supplementary material

Below is the link to the electronic supplementary material.

Supplementary Fig. S1. Comparisons of corneal thickness for 12-week adult mice of five genotypes. Each point is the mean of two measurements in the central cornea of a single eye (one eye per mouse) and the horizontal bars are group means for (A) the thickness of the whole central cornea, (B) the thickness of the central corneal stroma plus endothelium, (C) the thickness of the central corneal epithelium and (D) the number of corneal epithelial layers in the central cornea. The numbers of mice per group were 5 +*/*+, 5*fl*/+, 4*fl*/*fl*, 5 +*/*− and 5*fl*/−. A–C were analysed by 1-way analysis of variance (ANOVA; results shown on figure) and Tukey’s multiple comparison tests. D was analysed by a non-parametric Kruskal-Wallis (KW) test (shown on figure) and Dunn’s multiple comparison tests. Genotypes, with only different letters above the scatter plots, differ significantly (*P* < 0.05). Genotypes, with any shared letters above the scatter plots, do not differ significantly. Sexes are coloured differently (red, female; blue, male) but were not analysed separately. (PDF 114 kb)
