# Supplementary material for: Analysis of compound heterozygotes reveals that the mouse floxed Pax6tm1Ued allele produces abnormal eye phenotypes
Source: Transgenic Res. 2016 May 30;25(5):679–92. doi: 10.1007/s11248-016-9962-4 (PMC5023747; doi:10.1007/s11248-016-9962-4)
Supplement: Supplementary file 1 — Supplementary Fig. S1. Comparisons of corneal thickness for 12-week adult mice of five genotypes. Each point is the mean of two measurements in the central cornea of a single eye (one eye per mouse) and the horizontal bars are group means for (A) the thickness of the whole central cornea, (B) the thickness of the central corneal stroma plus endothelium, (C) the thickness of the central corneal epithelium and (D) the number of corneal epithelial layers in the central cornea. The numbers of mice per group were 5 +/+, 5fl/+, 4fl/fl, 5 +/− and 5fl/−. A–C were analysed by 1-way analysis of variance (ANOVA; results shown on figure) and Tukey’s multiple comparison tests. D was analysed by a non-parametric Kruskal-Wallis (KW) test (shown on figure) and Dunn’s multiple comparison tests. Genotypes, with only different letters above the scatter plots, differ significantly (P < 0.05). Genotypes, with any shared letters above the scatter plots, do not differ significantly. Sexes are coloured differently (red, female; blue, male) but were not analysed separately. (PDF 114 kb) [file 11248_2016_9962_MOESM1_ESM.pdf]

Analysis of compound heterozygotes reveals that the mouse floxed *Pax6*^{tm1Ued} allele produces abnormal eye phenotypes. Transgenic Research.

Dorà NJ, Crookshanks AJF, Leung KKY, Simpson YI, Mason JO, Price DJ and West JD.
Corresponding author: Dr. John West, University of Edinburgh. John.West@ed.ac.uk

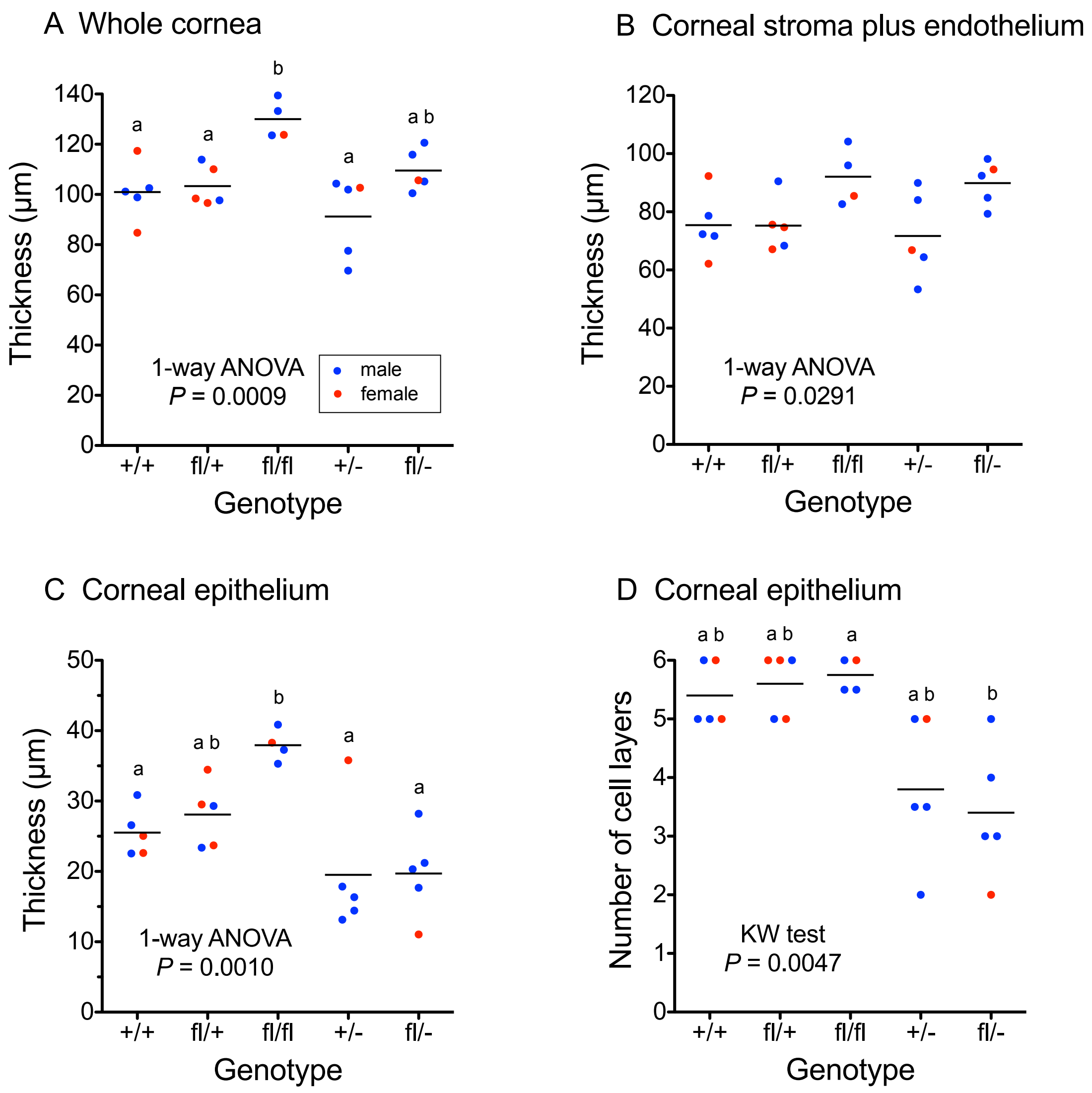

Supplementary Fig. S1. Comparisons of corneal thickness for 12-week adult mice of five genotypes. Each point is the mean of two measurements in the central cornea of a single eye (one eye per mouse) and the horizontal bars are group means for (A) the thickness of the whole central cornea, (B) the thickness of the central corneal stroma plus endothelium, (C) the thickness of the central corneal epithelium and (D) the number of corneal epithelial layers in the central cornea. The numbers of mice per group were 5 +/+, 5 *fl*/+, 4 *fl*/*fl*, 5 +/- and 5 *fl*/-. A-C were analysed by 1-way analysis of variance (ANOVA; results shown on figure) and Tukey's multiple comparison tests. D was analysed by a non-parametric Kruskal-Wallis (KW) test (shown on figure) and Dunn's multiple comparison tests. Genotypes, with only different letters above the scatter plots, differ significantly ($P <$ 0.05). Genotypes, with any shared letters above the scatter plots, do not differ significantly. Sexes are coloured differently (red, female; blue, male) but were not analysed separately.
